# Feasibility of an Ingestible Sensor-Based System for Monitoring Adherence to Tuberculosis Therapy

**DOI:** 10.1371/journal.pone.0053373

**Published:** 2013-01-07

**Authors:** Robert Belknap, Steve Weis, Andrew Brookens, Kit Yee Au-Yeung, Greg Moon, Lorenzo DiCarlo, Randall Reves

**Affiliations:** 1 Denver Public Health, Denver Health and Hospital Authority, Denver, Colorado, United States of America; 2 University of North Texas Health Science Center, Fort Worth, Texas, United States of America; 3 Department of Medicine, University of Washington, Seattle, Washington, United States of America; 4 Proteus Digital Health, Inc., Redwood City, California, United States of America; McGill University, Canada

## Abstract

Poor adherence to tuberculosis (TB) treatment hinders the individual’s recovery and threatens public health. Currently, directly observed therapy (DOT) is the standard of care; however, high sustaining costs limit its availability, creating a need for more practical adherence confirmation methods. Techniques such as video monitoring and devices to time-register the opening of pill bottles are unable to confirm actual medication ingestions. A novel approach developed by Proteus Digital Health, Inc. consists of an ingestible sensor and an on-body wearable sensor; together, they electronically confirm unique ingestions and record the date/time of the ingestion. A feasibility study using an early prototype was conducted in active TB patients to determine the system’s accuracy and safety in confirming co-ingestion of TB medications with sensors. Thirty patients completed 10 DOT visits and 1,080 co-ingestion events; the system showed 95^.^0% (95% CI 93^.^5–96^.^2%) positive detection accuracy, defined as the number of detected sensors divided by the number of transmission capable sensors administered. The specificity was 99^.^7% [95% CI 99^.^2–99^.^9%] based on three false signals recorded by receivers. The system’s identification accuracy, defined as the number of correctly identified ingestible sensors divided by the number of sensors detected, was 100%. Of 11 adverse events, four were deemed related or possibly related to the device; three mild skin rashes and one complaint of nausea. The system’s positive detection accuracy was not affected by the subjects’ Body Mass Index (p = 0^.^7309). Study results suggest the system is capable of correctly identifying ingestible sensors with high accuracy, poses a low risk to users, and may have high patient acceptance. The system has the potential to confirm medication specific treatment compliance on a dose-by-dose basis. When coupled with mobile technology, the system could allow wirelessly observed therapy (WOT) for monitoring TB treatment as a replacement for DOT.

## Introduction

Successfully curing tuberculosis (TB) requires that patients adhere to a multi-drug regimen for a minimum of 6 months [1]. Failure to take the medications correctly leads to drug-resistance, treatment failure, and TB transmission which threaten public health. These consequences were demonstrated in New York City in the late 1980s when nearly half of the patients started on therapy failed to complete treatment and the rates of both drug-susceptible and multi-drug resistant (MDR) TB increased [2]. Recently, similar events in Africa have led to a rise of extensively-drug resistant (XDR) TB, a nearly untreatable form of the disease [3]. The public health consequences of non-adherence to active TB treatment led to directly observed therapy (DOT) becoming the standard of care in the U.S. and around the world [1], [4]–[6].

Nevertheless, DOT is not feasible in much of the world, including parts of the U.S., due to cost constraints or long distances between patients and providers. Programs are forced to rely on modified DOT or self-administered therapy (SAT) where completion rates are worse. Adapting technology to monitor adherence has been studied using medication event monitoring devices which generally record the date and time a pill bottle is opened [7]. These devices do not document that a medication was ingested and until recently have been limited by their inability to provide rapid adherence information to clinicians [8]. Video phones have also been used as a substitute for in-person DOT [9]. While cost effective compared to direct encounters, they require a scheduled time for video observation and cannot ensure that medications are swallowed rather than hidden in the mouth to be discarded later. Both technologies have a very limited capability for detecting selective drug adherence where a patient chooses to take some but not all of their medications. Selective drug adherence is particularly concerning for TB as it increases the risk of acquired drug resistance.

A novel system using ingestible sensors to document medication adherence has been developed by Proteus Digital Health, Inc. Redwood City, California. Initial studies in animals and healthy volunteers demonstrated safety of the ingestible sensor system. The present study was designed to evaluate the accuracy, safety, and acceptability of the system in patients receiving active TB treatment by DOT. Limited data from this study was published previously as part of an aggregate report that combined the results from healthy volunteers and other small cohorts [10]. Here we present the results of the feasibility study in active TB treatment using an early prototype of the ingestible sensor system for electronically confirming medication ingestions.

## Materials and Methods

### Ethics Statement

The study was approved by the Western Institutional Review Board, Colorado Multiple Institutional Review Board (COMIRB), and the institutional review board at the University of North Texas.

This was a prospective, non-randomized, descriptive study of the safety, performance and acceptability of an ingestible sensor system in patients with active TB. The study was conducted at 2 sites, 1 each in Denver, Colorado and Fort Worth, Texas. Written informed consent was obtained from all participants. Patients aged 18 years or older taking active TB treatment were eligible to participate if they had received more than 10 days of medications for suspected or confirmed TB. Exclusion criteria included pregnancy, acute gastrointestinal (GI) symptoms, history of major GI surgery, end-stage liver or kidney disease, presence of an implanted electronic medical device, current alcohol or drug abuse that could impair follow-up, known allergies to any substances that could compromise patient safety, and recent participation in another medical device study.

### The Ingestible Sensor System

The system consists of a 1.0 mm×1.0 mm ingestible sensor and an on-body wearable sensor. The ingestible sensors are activated by gastric fluids, independent of the acidity level, and communicate unique identifying signatures to the body surface. The system uses a conductive method of communication and not radio-frequency which ensures the information is confined to the body of the user, thus preserving privacy. The on-body sensor counts the number of times each unique signature is received. For this study, the ingestible sensors were attached to inert tablets and co-ingested with the TB medications. The system recorded the date and time of an ingestion event after a unique signature was received ≥10 times. The ingestible sensors are designed to communicate for approximately 7 minutes after which they are inactive and get eliminated in the feces. The system is capable of identifying and differentiating among multiple simultaneously ingested sensors.

### Study Objectives

The primary objective was to determine the detection accuracy of the ingestible sensor system when co-administered under direct observation with active TB medications. This included the ability to correctly register when ingestions occurred (positive detection accuracy) and the ability to correctly identify the unique signatures of multiple sensors ingested simultaneously (identification accuracy). A secondary objective was to measure whether the wearable sensor would detect any false signatures. Other secondary objectives were monitoring for adverse events and obtaining feedback from providers and participants.

### Study Procedure

Each participant co-ingested two inert tablets with their TB medications during 10 consecutive DOT visits. Of the 20 inert tablets, 18 carried two ingestible sensors affixed on opposite sides and two tablets were “dummies” with no active sensors. After informed consent, study staff conducted a brief clinical history and physical exam. Participants were asked about new symptoms at each study visit, but no laboratory tests were conducted for the purposes of this study. There were no restrictions on diet during the study and dietary information was not collected.

At each visit, a wearable sensor prototype containing a compact flash card was attached via four body surface electrodes. Patients were asked to ingest their TB medications and the inert tablets directly from cups with at least 4 oz. of water while study staff observed. They were then required to wear the on-body sensor prototype for 30 minutes after the ingestion but were allowed to move around freely. After two study visits at the clinic, patients and investigators could choose to complete the remaining eight visits at the patient’s home using the same procedures. A final follow-up visit was conducted two weeks after the last co-ingestion event at which time participants were asked about changes in their health and asked to provide feedback on the system. Patients were compensated per protocol for their participation in the study.

### Statistical Analysis

The sample size calculation was done assuming a 94% detection rate with the goal of demonstrating a narrow confidence interval around the positive detection accuracy for a lower confidence boundary of 90%. Thirty participants were included representing a convenience sample of patients who met enrollment criteria.

Baseline demographics and other characteristics including weight, height, and body mass index (BMI) were analyzed. Descriptive statistics are presented for continuous variables and frequency distributions for categorical variables. The primary outcome measures of system performance were the positive detection accuracy, defined as the number of ingestible sensors detected divided by those ingested, and the identification accuracy, defined as the number of ingestible sensors identified with a correct signature divided by those detected. A secondary outcome included determining whether any false signatures would be detected either when the “dummy” tablets were ingested or at any other time.

The positive detection accuracy and identification accuracy were calculated including the 95% confidence intervals for all ingestible sensors both by subject and across subjects. The positive detection accuracy across subjects was also determined using a mixed model for repeated measures with terms for DOT visit day and number of ingestible sensors as fixed effects, and subject as a random effect. The association of each covariate and the measures of system performance were assessed using the F-test from the mixed model for repeated measures. Also, the effect of BMI as a covariate on system performance was investigated.

## Results

Forty-nine subjects were screened and 30 enrolled, 19 in Fort Worth and 11 in Denver. The most frequent reason patients declined enrollment was difficulty with the time commitment required for the study. All 30 participants completed ten study visits each for a total of 300 DOT visits. Their demographics are listed in Table 1, and the TB treatment regimens are listed in Table 2. Twenty-one of 30 (70%) patients were taking non-TB medications for concurrent diseases.

**Table 1:** Characteristics of the study population.

Category	Subcategory	Enrolled = 30
		N (%)
Age in years, median (range)		44 (22–79)
Sex	Female	14 (47)
Race/Ethnicity		
	Black	9 (30)
	Hispanic	13 (43)
	Non-Hispanic, White	3 (10)
	Asian	3 (10)
	Other	2 (7)
Foreign Born		19 (63)
Weight in pounds, median (range)		135 (93·0–188·0)
Body Mass Index kg/m^2^, mean (range)		25·5 (16·0–31·1)
Concurrent Illness		
	Diabetes	4 (13)
	HIV Infection	3 (10)
Taking non-TB Medications		21 (70)

**Table 2:** TB treatment regimens received during study visits.

Tuberculosis Regimen*	# of subjectsn(%)
INH, Rif, PZA, EMB	11
INH, Rif, PZA, EMB, Moxi	3
INH, Rif	3
Rifabutin, Moxi, EMB	3
INH, Rif, PZA	3
INH, Rif, EMB	2
INH, Rifabutin, PZA	1
INH, PZA, EMB, Moxi	1
INH, Rifabutin, EMB, Moxi	1
Rifampin, EMB, Moxi	1
Rifampin, Moxi	1

* INH = isoniazid, Rif = rifampin, PZA = pyrazinamide, EMB = ethambutol, Moxi = moxifloxacin.

A total of 1,080 sensors were co-ingested with TB medications during the study. The wearable sensor prototypes malfunctioned in nine of the visits (34/1080 sensor ingestions) resulting in 3% of data missing, and were attributed to improper insertion of the compact flash cards for data recording. Of the remaining 1,046 ingestible sensors for which data was recorded, 1,026 were detected. The primary analysis considered the ingestions to be independent observations and treated the missing data as detection failures, resulting in a 95.0% positive detection accuracy (95% CI = 93^.^5–96^.^2%). If the missing records were excluded, the system’s positive detection accuracy was 98.1% (95% CI 97^.^1–98^.^8%), and using a repeated measures model, it increased to 99.6% (95% CI 98^.^6–100).

Patients were stratified into 3 groups based on BMI, <18^.^5 (n = 4), 18^.^5–25 (n = 16), and >25 (n = 9). No difference was observed in the positive detection accuracy among these groups (p = 0^.^7309).

Each ingestible sensor communicated a unique signature and the identification accuracy of the system was determined. Of the 1,026 sensors detected during the study, all were correctly identified by their unique signature. The identification accuracy was therefore 100% with a 95% confidence interval of 99^.^6–100%.

A secondary objective was to assess whether false positive signatures would be detected. This was evaluated in 2 ways. First, each participant ingested 2 placebo tablets without ingestible sensors attached during the study. None of the placebos produced a false positive signature. Next, signatures recorded from all study visits were compared against the known signatures of the sensors ingested and the specificity was calculated in a post-hoc analysis. There were 3 signatures recorded that did not correlate with a sensor ingestion, resulting in a specificity of 99^.^7% [95% CI 99^.^2–99^.^9%]. These false ingestions were recorded based on signatures that were detected between 13 and 15 times while the average number of signatures for true sensors was 733 (range 11 to 2,673).

Perceptions of the system prototype were assessed using a post-study questionnaire. Questions were answered using a Likert scale from 1 (strongly agree) to 10 (strongly disagree). Participants were also encouraged to provide qualitative feedback about their experience using the system. Twenty-four of 30 participants completed evaluable post-study questionnaires. Three patients missed their follow-up appointment, 2 questionnaires were not interpretable, and 1 could not be completed due to an IRB delay. Of the 24 evaluable questionnaires, 20 (83%) said they would be comfortable using the system every day, and 18 (75%) would be comfortable using it long term (>3months). Most, 20 (83%) felt the clinic should offer the system to TB patients if it were available.

There were a total of 11 adverse events reported in eight participants. Three were considered device related and described as mild skin rashes at the site where a body surface electrode was located. One participant had nausea that was recorded as possibly study-related. The only serious adverse event was a hospitalization for community-acquired pneumonia that resolved with antibiotics and was considered unrelated to the study or device.

## Discussion

Adherence to self-administered TB treatment (SAT) has been studied since the advent of combination therapy in the 1950’s [11]–[14]. Most studies concluded that the overall adherence to treatment is poor and that it is difficult to predict non-adherence based on clinical or demographic factors. Consequently, DOT has yielded the greatest success for TB treatment completion, particularly when combined with a patient-centered approach in which barriers to adherence are promptly identified and addressed. DOT is considered the standard of care for treating TB patients worldwide [1], [4], [5].

However, the cost and personnel required to sustain a high quality DOT program have prevented universal implementation. A study in San Francisco found that 60% of TB patients between 1998 and 2000 received SAT at the start of treatment and these patients had worse outcomes, including death and relapse, when compared with patients started on DOT [15]. Still only 56% of newly diagnosed TB patients in the U.S. received treatment entirely by DOT in 2008, the most recent year with data reported [16]. Consequently, cost-effective alternatives to DOT that enable clinicians to monitor real-time adherence are needed.

The ingestible sensor system is a novel approach for electronically documenting the ingestion of medications. In this early clinical feasibility study, the system appears to be effective at accurately detecting the ingestion of multiple sensors attached to inert tablets when co-administered with active TB medications. Three false positive detections occurred and were likely from environmental noise or an interfering signature from the simultaneous ingestion of multiple sensors. Increasing the number of signatures required to record an ingestion event would eliminate false positives but could have a small impact on the overall sensitivity.

There are several limitations to this study. The sample size for this feasibility study was small and larger studies are needed to further document the sensitivity, specificity, usability, acceptability, and cost-effectiveness of the system. The patients enrolled represented a convenience sample from 2 sites and were not randomized or blinded. Also, only 24 of 30 patients completed evaluable post-study questionnaires. Therefore, the qualitative feedback may not be representative of the larger population of TB patients. Finally, our study used an early prototype of wearable sensor that resulted in missing data for 3% of study visits. A newer version of the wearable sensor has been developed and will need to be evaluated in future clinical trials for both performance and patient acceptability.

**Figure 1:**
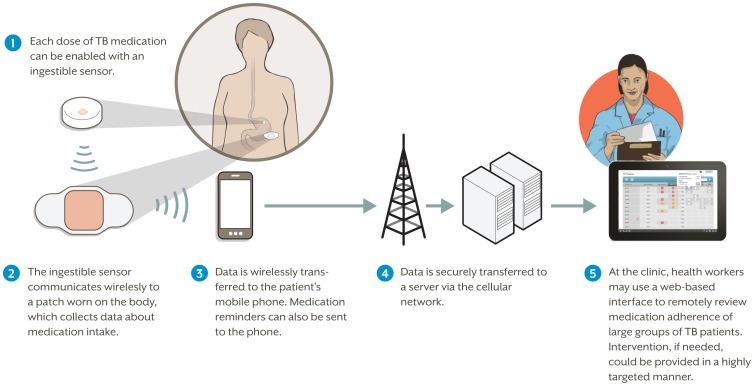
Overview of the future, fully developed system to support Wirelessly Observed Therapy (WOT).

As shown in Figure 1, current enhancements to the system include the development of a patch that can be worn for up to a week. Future plans include transmitting the data using mobile technology to allow treatment monitoring termed wirelessly observed therapy (WOT) that will provide patients and clinicians with real-time access to the data. Ultimately, implementation of this technology will require co-formulation or co-encapsulation with the treatment medications, which was not feasible at the time of this proof-of-concept study.

This study supports the concept of monitoring TB treatment adherence using ingestible sensors administered with TB medications. A recent cost-analysis that modeled WOT compared to DOT found that WOT would cost less for TB control programs [17]. The future application of the system could allow more efficient use of resources, particularly personnel, and facilitate the expansion of monitored TB treatment into more distant and previously difficult to reach populations. As with current DOT programs, success depends upon promptly intervening when poor adherence is identified.
